# Positive solutions of fractional integral equations by the technique of measure of noncompactness

**DOI:** 10.1186/s13660-017-1497-6

**Published:** 2017-09-15

**Authors:** Hemant Kumar Nashine, Reza Arab, Ravi P Agarwal, Manuel De la Sen

**Affiliations:** 1 Department of Mathematics, Texas A&M University, Kingsville, Texas 78363-8202 USA; 2 Department of Mathematics, Sari Branch, Islamic Azad University, Sari, Iran; 3 Institute of Research and Development of Processes IIDP Faculty of Science and Technology, University of the Basque Country, Leioa, Spain

**Keywords:** 47H10, 54H25, measures of noncompactness, Darbo’s fixed point theorem, coupled fixed point, integral equations

## Abstract

In the present study, we work on the problem of the existence of positive solutions of fractional integral equations by means of measures of noncompactness in association with Darbo’s fixed point theorem. To achieve the goal, we first establish new fixed point theorems using a new contractive condition of the measure of noncompactness in Banach spaces. By doing this we generalize Darbo’s fixed point theorem along with some recent results of (Aghajani *et al.* (J. Comput. Appl. Math. 260:67-77, 2014)), (Aghajani *et al.* (Bull. Belg. Math. Soc. Simon Stevin 20(2):345-358, 2013)), (Arab (Mediterr. J. Math. 13(2):759-773, 2016)), (Banaś *et al.* (Dyn. Syst. Appl. 18:251-264, 2009)), and (Samadi *et al.* (Abstr. Appl. Anal. 2014:852324, 2014)). We also derive corresponding coupled fixed point results. Finally, we give an illustrative example to verify the effectiveness and applicability of our results.

## Introduction

Fractional calculus is the study of integrals and derivatives of an arbitrary order. Fractional calculus seeks to find the integrals and derivatives of a real or even complex order using the Gamma function, Euler’s generalization of the factorials. In modern times differential/integral equations with nonintegral order have drawn the attention of numerous researchers due to their wide applications in several fields of science and engineering. The need for fractional order differential/integral equations stems in part from the fact that many phenomena cannot be modeled by differential/integral equations with integer derivatives. Analytical and numerical techniques have been implemented to study such equations.

Due to the importance of fractional calculus, it is necessary to discuss the related problems and work on them. In this work, we study the problem of the existence of positive solutions for integral equations of the form

1.1 $$\begin{aligned} &x(t)=a(t)+\frac{h(t,x(t))}{\Gamma(\gamma)} \int_{0}^{t}\frac {f^{\prime}(s)}{(f(t)-f(s))^{1-\gamma}}g \bigl(t,s,x(s) \bigr) \,ds, \\ &\quad t\in I=[0,1], 0< \gamma< 1, \end{aligned}$$

where $\Gamma(\cdot)$ is the (Euler) Gamma function defined by $\Gamma (\gamma)=\int_{0}^{\infty}t^{\gamma-1}e^{-t}\,dt$. Let us recall that the function $h(t,x)$ involved in equation (1.1) generates the superposition operator *H* defined by the formula $(Hx)(t)=h(t,x(t))$, where $x=x(t)$ is an arbitrary function defined on *I* (*cf.* [6, 7]). We are going to show that equation (1.1) has a positive solution that belongs to space $C_{+}(I)=C([0,1]; \mathbb{R}_{+})$. The obtained results extend several papers (see [1–5, 8], for example). Finally, an example is presented to show the efficiency of our results.

## Preliminaries

Throughout this paper, we assume that $(E, \Vert \cdot \Vert )$ is a real Banach space with zero element 0. Let $\mathbb{R}=(-\infty, +\infty), \mathbb{R}^{+}=[0, +\infty)$, and $\mathbb{N}=\{1,2,3,\ldots\}$. Let $\overline{B}(x,r)$ denote the closed ball centered at *x* with radius *r*. The symbol $\overline{B}_{r}$ stands for the ball $\overline{B}(0,r)$. For *X*, a nonempty subset of *E*, we denote by *X̅* and Conv*X* the closure and the closed convex hull of *X*, respectively. Moreover, let us denote by ${\mathfrak{M}}_{E}$ the family of nonempty bounded subsets of *E* and by ${\mathfrak{N}}_{E}$ its subfamily consisting of all relatively compact subsets of *E*. We use the following definition of measure of noncompactness (MNC, for short) given in [4].

### Definition 2.1

[4]

A mapping $\beta:\mathfrak{M}_{E} \longrightarrow\mathbb{R}_{+}$ is said to be an MNC in *E* if it satisfies the following conditions:

(1^∘^): the family $\operatorname{ker}\beta=\{X\in\mathfrak{M}_{E}: \beta(X)=0\}$ is nonempty and $\operatorname{ker}\beta\subset\mathfrak{N}_{E}$,
(2^∘^): $X\subset Y\Longrightarrow\beta(X)\leq\beta(Y)$,
(3^∘^): $\beta(\overline{X})=\beta(X)$,
(4^∘^): $\beta(\operatorname{Conv} X) = \beta(X)$,
(5^∘^): $\beta(\lambda X + (1 - \lambda)Y)\leq\lambda\beta (X) + (1 -\lambda)\beta(Y)$ for $\lambda\in[0, 1]$,
(6^∘^): if $\{X_{n}\}$ is a sequence of closed sets from $\mathfrak{M}_{E}$ such that $X_{n+1} \subset X_{n}$ for $n=1,2,\ldots $ and if $\lim_{n\rightarrow\infty} \beta(X_{n}) = 0$, then the intersection set $X_{\infty}= \bigcap_{n=1}^{\infty}X_{n}$ is nonempty.

The subfamily ker*β*, defined by (1^∘^), represents the kernel of the MNC *β* and since

$$\beta(X_{\infty})=\beta \Biggl(\bigcap_{n=1}^{\infty}X_{n} \Biggr)\leq\beta(X_{n}), $$

we see that

$$\beta \Biggl(\bigcap_{n=1}^{\infty}X_{n} \Biggr)=0. $$

Therefore, $X_{\infty} \in \operatorname{ker} \beta$.

From now on we denote by *β* an MNC and *C* to be a nonempty, bounded, closed and convex subset of a Banach space *E*.

Darbo’s fixed point theorem (DFPT) is a very important generalization of Schauder’s fixed point theorem and Banach’s fixed point theorem.

### Lemma 2.2

Banaś and Goebel [9]

*Let* *Q* *be a self*-*continuous operator on* *C* *and assume* ∃ *to be a constant* $k\in[0, 1)$ *such that*

$$\beta \bigl(Q(S) \bigr)\leq k\beta(S), $$

*for any nonempty subset* $S\subset C$. *Then* *Q* *has at least one fixed point in* *C*.

Following the trend of Lemma 2.2, various authors proved several DFPTs and their coupled versions by using different types of contractive conditions in the sense of the MNC (for example, see [1–5, 8]).

## New fixed point theorem for shifting distance functions

To complete the proof, we need the following notions.

### Definition 3.1

[10]

Let $\Psi, \Phi: [0,\infty)\longrightarrow\Bbb{R}$ be two functions. The pair of functions $(\Psi,\Phi)$ is said to be a pair of shifting distance functions if the following conditions hold:

1. For $u, v \in[0,+\infty)$, if $\Psi(u)\leq\Phi(v)$, then $u\leq v$.
2. For $u_{n}, v_{n} \in[0,+\infty)$, with
  $$\lim_{n \rightarrow\infty}u_{n}=\lim_{n \rightarrow\infty}v_{n}=w, $$
  if $\Psi(u_{n})\leq\Phi(v_{n})$ for all *n*, then $w=0$.

We denote by ϒ a pair $(\Psi,\Phi)$ of shifting distance functions.

### Example

[10]

Take $\Psi(t)=\ln(\frac{1+2t}{2})$, $\Phi (t)=\ln(\frac{1+t}{2})$ for all $t \in[0,\infty)$. Then obviously they belong to ϒ.

### Definition 3.2

[11]

Let Δ be a set of functions $\chi: \mathbb{R}^{+} \rightarrow[0, 1)$ satisfying

$$\chi(t_{n})\rightarrow\quad\Rightarrow\quad t_{n} \rightarrow0. $$

Let $\Bbb{F}$ be class of functions $F: [0,\infty) \times[0,\infty )\longrightarrow[0,\infty)$ satisfying the following conditions:

(i) $\max\lbrace a,b \rbrace\leq F(a,b)$ for $a,b\geq0$,
(ii) *F* is continuous,
(iii) $F(a+b, c+d) \leq F(a,c)+F(b,d)$.

We are now in a position to state and prove a new DFPT theorem.

### Theorem 3.3

*Let* *T* *be a self*-*continuous operator on* *C* *such that*

3.1 $$\begin{aligned} \Psi \bigl(F \bigl(\beta \bigl(T(X) \bigr),\varphi \bigl(\beta(TX) \bigr) \bigr) \bigr) \leq\Phi \bigl(F \bigl(\beta(X),\varphi \bigl(\beta(X) \bigr) \bigr) \bigr), \end{aligned}$$

*for any subset* *X* *of* *C*, *where* $F\in\Bbb{F}$, $(\Psi,\Phi) \in \Upsilon$, *and* $\varphi: \Bbb{R}_{+}\longrightarrow\Bbb{R}_{+}$ *is a continuous function*. *Then* *T* *has at least one fixed point in* *C*.

### Proof

We start with $C_{0}=C$ and construct a sequence $\{C_{n}\}$ such that $C_{n+1}=\operatorname{Conv}(TC_{n})$, for $n\geq0$. $TC_{0}=TC\subseteq C=C_{0}, C_{1}=\operatorname{Conv}(TC_{0})\subseteq C=C_{0}$. Therefore by continuing this process we have

$$\begin{aligned} C_{0}\supseteq C_{1}\supseteq\cdots\supseteq C_{n}\supseteq C_{n+1} \supseteq\cdots. \end{aligned}$$

If ∃ a natural number *N* such that $\beta(C_{N})=0$, then $C_{N}$ is compact and concludes the result through Schauder’s fixed point theorem. So we consider $\beta(C_{n})> 0$ for $n\geq0$. Also, by (3.1), we have

3.2 $$\begin{aligned} \Psi \bigl(F \bigl(\beta(C_{n+1}), \varphi \bigl(\beta(C_{n+1}) \bigr) \bigr) \bigr) &=\Psi \bigl(F \bigl(\beta \bigl(\operatorname{Conv}(TC_{n}) \bigr),\varphi \bigl(\beta \bigl( \operatorname{Conv}(TC_{n}) \bigr) \bigr) \bigr) \bigr) \\ &=\Psi \bigl(F \bigl(\beta(TC_{n}),\varphi \bigl( \beta(TC_{n}) \bigr) \bigr) \bigr) \\ &\leq \Phi \bigl[F \bigl(\beta(C_{n}),\varphi \bigl( \beta(C_{n}) \bigr) \bigr) \bigr]. \end{aligned}$$

This implies that $\lbrace F(\beta(C_{n}),\varphi(\beta(C_{n}))) \rbrace $ is a nonincreasing sequence of positive real numbers by $(1)$ of Definition 3.1. Hence, there is an $r\geq0$ such that

$$\lim_{n \rightarrow\infty}F \bigl(\beta(C_{n}),\varphi \bigl( \beta(C_{n}) \bigr) \bigr)=\lim_{n \rightarrow\infty}F \bigl( \beta(C_{n+1}),\varphi \bigl(\beta(C_{n+1}) \bigr) \bigr)=r. $$

Then, in view of (3.2) and (2) of Definition 3.1, we get $r=0$ and hence

3.3 $$\begin{aligned} \lim_{n \rightarrow\infty}F \bigl(\beta(C_{n}), \varphi \bigl(\beta(C_{n}) \bigr) \bigr)=0. \end{aligned}$$

Using (3.3) and (i) of property $\Bbb{F}$, we get

$$\lim_{n \rightarrow\infty}\beta(C_{n})=\lim_{n \rightarrow\infty } \varphi \bigl(\beta(C_{n}) \bigr)=0. $$

Now since $C_{n}$ is a nested sequence, in view of (6^∘^) of Definition 2.1, we conclude that $C_{\infty}= \bigcap_{n=1}^{\infty}C_{n}$ is a nonempty, closed, and convex subset of *C*. Besides we know that $C_{\infty}$ belongs to ker*β*. So $C_{\infty }$ is compact and invariant under the mapping *T*. Consequently, Schauder’s fixed point theorem implies this result in $C_{\infty}$, but as $C_{\infty}\subset C$, the result is true in *C*. □

Taking $F(a,b)=a+b$ in Theorem 3.3, we obtain the following.

### Theorem 3.4

*Let* *T* *be a self*-*continuous operator on* *C* *such that*

3.4 $$\begin{aligned} \Psi \bigl(\beta \bigl(T(X) \bigr)+\varphi \bigl(\beta(TX) \bigr) \bigr) \leq\Phi \bigl(\beta(X)+\varphi \bigl(\beta(X) \bigr) \bigr), \end{aligned}$$

*for any subset* *X* *of* *C*, *where* $(\Psi,\Phi) \in\Upsilon$ *and* $\varphi : \Bbb{R}_{+}\longrightarrow\Bbb{R}_{+}$ *is a continuous function*. *Then* *T* *has at least one fixed point in* *C*.

### Remark 3.5

Take $\varphi\equiv0$ in Theorem 3.4. Then Theorem 6 of [5] is obtained.

### Remark 3.6

Take $\varphi\equiv0, \Psi(t)=t, \Phi(t)=\lambda t$ for $t\geq0$ and $\lambda\in[0,1)$ in Theorem 3.4. Thus we get DFPT.

### Remark 3.7

Take $\Psi(t)=t, \Phi(t)=\lambda t$ for $t\geq0$ and $\lambda\in [0,1)$ in Theorem 3.4. We get Theorem 5 of [8].

### Remark 3.8

Set $\varphi\equiv0, \Psi(t)=t, t\geq0$ and let the function Φ satisfy $\lim_{n \rightarrow\infty}\Phi^{n}(t)=0$ for any $t\geq0$ in Theorem 3.4. Then we get Theorem 2.2 of [2].

Taking $\Psi=I$ in Theorem 3.3, we have the following result.

### Corollary 3.9

*Let* *T* *be a self*-*continuous operator on* *C* *such that*

$$\begin{aligned} F \bigl(\beta \bigl(T(X) \bigr),\varphi \bigl(\beta(TX) \bigr) \bigr) \leq\Phi \bigl(F \bigl(\beta(X),\varphi \bigl(\beta(X) \bigr) \bigr) \bigr), \end{aligned}$$

*for any subset* *X* *of* *C*, *where* $F\in\Bbb{F}$ *and* $\varphi: \Bbb {R}_{+}\longrightarrow\Bbb{R}_{+}$ *is a continuous function*, *where* $\Phi:[0,\infty) \rightarrow\Bbb{R}$ *is a function such that*

- *for* $u, v \in[0,+\infty)$, *if* $u\leq\Phi(v)$, *then* $u\leq v$,
- *for* $u_{n}, v_{n} \in[0,+\infty)$ *with*
  $$\lim_{n \rightarrow\infty}u_{n}=\lim_{n \rightarrow\infty}v_{n}=w, $$
  *if* $u_{n}\leq\Phi(v_{n})$ *for all* *n*, *then* $w=0$.

*Then* *T* *has at least one fixed point in* *C*.

### Remark 3.10

Take $F(a,b)=a+b$ in Corollary 3.9. We get Theorem 3.5 of [3].

Following Proposition 9 (see [10]) and Theorem 3.3, we conclude the following.

### Theorem 3.11

*Let* *T* *be a self*-*continuous operator on* *C* *such that*

$$\begin{aligned} \psi \bigl(F \bigl(\beta \bigl(T(X) \bigr),\varphi \bigl(\beta(TX) \bigr) \bigr) \bigr) \leq\psi \bigl(F \bigl(\beta(X),\varphi \bigl(\beta(X) \bigr) \bigr) \bigr)-\phi \bigl(F \bigl(\beta(X),\varphi \bigl(\beta(X) \bigr) \bigr) \bigr), \end{aligned}$$

*for any subset* *X* *of* *C*, *where* $F\in\Bbb{F}$, $\varphi: \Bbb {R}_{+}\longrightarrow\Bbb{R}_{+}$ *is a continuous function*, *and* $\psi ,\phi:[0,\infty) \rightarrow[0,\infty)$ *are two nondecreasing and continuous functions satisfying* $\psi(t)=\phi(t)=0$ *if and only if* $t=0$. *Then* *T* *has at least one fixed point in* *C*.

### Remark 3.12

Take $\varphi\equiv0$ and $F(a,b)=a+b$ in Theorem 3.11. Then Theorem 2.1 of [2] is obtained.

### Remark 3.13

Take $F(a,b)=a+b$ in Theorem 3.11. We get Theorem 6 of [8].

### Theorem 3.14

*Let* *T* *be a self*-*continuous operator on* *C* *such that*

$$\begin{aligned} F \bigl(\beta \bigl(T(X) \bigr),\varphi \bigl(\beta(TX) \bigr) \bigr) \leq\chi \bigl(F \bigl(\beta(X),\varphi \bigl(\beta (X) \bigr) \bigr) \bigr) F \bigl( \beta(X),\varphi \bigl(\beta(X) \bigr) \bigr), \end{aligned}$$

*for any subset* *X* *of* *C*, *where* $F\in\Bbb{F}$ *and* $\chi\in\Delta$. *Then* *T* *has at least one fixed point in* *C*.

### Remark 3.15

Take $F(a,b)=a+b$ and $\varphi\equiv0$ in Theorem 3.14. We get Theorem 2.1 of [1].

## Coupled fixed point theorems

Here we derive some new coupled fixed point (CFP) results by means of the MNC.

### Definition 4.1

[12]

An element $(u, v) \in{E}^{2}$ is called a CFP of mapping ${G}: {E}^{2} \to{E}$ if ${G}(u, v) = u$ and ${G}(v, u) = v$.

The first CFP result is the following.

### Theorem 4.2

*Suppose that* ${G}: C^{2} \rightarrow C^{2}$ *is continuous operator such that*, *for* $i,j \in\{1,2\}, i \neq j$,

4.1 $$\begin{aligned} &\Psi\bigl(F \bigl(\beta \bigl({{G}}(X_{i} \times X_{j}) \bigr), \varphi \bigl(\beta \bigl({{G}}(X_{i} \times X_{j}) \bigr) \bigr) \bigr)\bigr) \\ &\quad\leq \frac{1}{2} \Phi \bigl(F \bigl( \beta(X_{i})+ \beta(X_{j}), \varphi \bigl(\beta (X_{i})+ \beta(X_{j}) \bigr) \bigr) \bigr) \end{aligned}$$

*for all* $(X_{i},X_{j}) \in C \times C $, *where* $F\in\Bbb{F}$, $\varphi: \Bbb {R}_{+}\longrightarrow\Bbb{R}_{+}$ *is a continuous sub*-*additive function*, *and* $(\Psi,\Phi) \in\Upsilon$ *are sub*-*additive functions*. *Then* *G* *has at least one CFP* $(u, v) \in C^{2}$.

### Proof

Consider the map ${{G}}: {X}^{2} \to{X}^{2}$ defined by the formula

$$\widehat{{G}}(u, v) = \bigl({{G}}(u, v), {{G}}(v, u) \bigr). $$

*Ĝ* is continuous due to the continuity of *G*. Following [13], we define a new MNC in the space ${X} \times{X}$ as

$$\widehat{{\beta}}({M}) = \beta({X}_{1}) + \beta({X}_{2}), $$

where ${X}_{i}$, $i = 1, 2$ denote the natural projections of *X*. Without loss of generality, we assume *M* is a nonempty subset of ${X}^{2}$. Hence, by condition (4.1) and using (2^∘^) of Definition 2.1 we conclude that

$$\begin{aligned} \widehat{{\beta}} \bigl(\widehat{{G}}({M}) \bigr) &\leq \widehat{\beta } \bigl({{G}}({X}_{1} \times{X}_{2}) \times{{G}}(X_{2} \times{X}_{1}) \bigr) \\ &= \beta \bigl({{G}}({X}_{1} \times{X}_{2}) \bigr)+\beta \bigl({{G}}({X}_{2} \times{X}_{1}) \bigr), \end{aligned}$$

which implies

$$\begin{aligned} &\Psi \bigl(F \bigl(\widehat{{\beta}} \bigl(\widehat{{G}}({M}) \bigr), \varphi \bigl(\widehat{{\beta }} \bigl(\widehat{{G}}({M}) \bigr) \bigr) \bigr) \bigr) \\ &\quad\leq \Psi \bigl(F \bigl(\widehat{\beta} \bigl({{G}}({X}_{1} \times{X}_{2}) \times {{G}}({X}_{2} \times{X}_{1}) \bigr), \varphi \bigl(\widehat{\beta} \bigl({{G}}({X}_{1} \times {X}_{2}) \times{{G}}({X}_{2} \times{X}_{1}) \bigr) \bigr) \bigr) \bigr) \\ &\quad\leq \Psi \bigl(F \bigl(\beta \bigl({{G}}({X}_{1} \times{X}_{2}) \bigr)+\beta \bigl({{G}}({X}_{2} \times {X}_{1}) \bigr), \varphi \bigl(\beta \bigl({{G}}({X}_{1} \times{X}_{2}) \bigr) \bigr)+ \varphi \bigl(\beta \bigl({{G}}({X}_{2} \times{X}_{1}) \bigr) \bigr) \bigr) \bigr) \\ &\quad\leq \Psi \bigl(F \bigl(\beta \bigl({{G}}({X}_{1} \times{X}_{2}) \bigr), \varphi \bigl(\beta \bigl({{G}}({X}_{1} \times{X}_{2}) \bigr) \bigr) \bigr) \\ &\qquad{}+ F \bigl(\beta \bigl({{G}}({X}_{2} \times{X}_{1}) \bigr), \varphi \bigl( \beta \bigl({{G}}({X}_{2} \times{X}_{1}) \bigr) \bigr) \bigr) \bigr) \\ &\quad\leq \Psi \bigl(F \bigl(\beta \bigl({{G}}({X}_{1} \times{X}_{2}) \bigr), \varphi \bigl(\beta \bigl({{G}}({X}_{1} \times{X}_{2}) \bigr) \bigr) \bigr) \bigr) \\ &\qquad{}+ \Psi \bigl(F \bigl(\beta \bigl({{G}}({X}_{2} \times {X}_{1}) \bigr), \varphi \bigl( \beta \bigl({{G}}({X}_{2} \times{X}_{1}) \bigr) \bigr) \bigr) \bigr) \\ &\quad\leq \frac{1}{2} \Phi \bigl(F \bigl(\beta({X}_{1})+ \beta({X}_{2}) \bigr), \varphi \bigl(\beta ({X}_{1})+ \beta({X}_{2}) \bigr) \bigr)\\ &\qquad{} + \frac{1}{2} \Phi \bigl(F \bigl( \beta({X}_{2})+\beta({X}_{1}), \varphi \bigl( \beta({X}_{2})+\beta({X}_{1}) \bigr) \bigr) \bigr) \\ &\quad= \Phi \bigl(F \bigl(\beta({X}_{1})+ \beta({X}_{2}), \varphi \bigl(\beta({X}_{1})+\beta ({X}_{2}) \bigr) \bigr) \bigr) \\ &\quad= \Phi \bigl(F \bigl(\widehat{\beta}({M}), \varphi \bigl(\widehat{ \beta}({M}) \bigr) \bigr) \bigr), \end{aligned}$$

that is,

$$\begin{aligned} \Psi \bigl(F \bigl(\widehat{\beta} \bigl(\widehat{{G}}({M}) \bigr), \varphi \bigl( \widehat{{\beta }} \bigl(\widehat{{G}}({M}) \bigr) \bigr) \bigr) \bigr)\leq \Phi \bigl(F \bigl(\widehat{\beta}({M}), \varphi \bigl(\widehat{\beta}({M}) \bigr) \bigr) \bigr). \end{aligned}$$

Following Theorem 3.3, *Ĝ* has at least one fixed point in ${X}^{2}$, and hence *G* has a CFP. □

The second outcome of this section is the following.

### Theorem 4.3

*Suppose that* ${G}: C^{2} \rightarrow C^{2} $ *is a continuous operator such that*, *for* $i,j \in\{1,2\}, i \neq j$,

4.2 $$\begin{aligned} \Psi \begin{pmatrix}F (\beta ({{G}}({X}_{i} \times{X}_{j}) ),\\ \varphi (\beta ({{G}}({X}_{i} \times{X}_{j}) ) ) ) \end{pmatrix} \leq\Phi \begin{pmatrix} F (\max \{\beta({X}_{i}),\beta({X}_{j}) \}, \\\varphi (\max \{ \beta({X}_{i}),\beta({X}_{j}) \} ) ) \end{pmatrix} \end{aligned}$$

*for all* $(X_{i},X_{j}) \in C \times C $, *where* $F\in\Bbb{F}$, $(\Psi,\Phi) \in\Upsilon$ *and* $\varphi: \Bbb{R}_{+}\longrightarrow\Bbb{R}_{+}$ *is a continuous function*. *Then* *G* *has at least one CFP* $(u, v) \in C^{2}$.

### Proof

Consider the map ${{G}}: {X} \times{X} \to{X} \times{X}$, defined by the formula

$$\widehat{{G}}(u, v) = \bigl({{G}}(u, v), {{G}}(v, u) \bigr). $$

*Ĝ* is continuous due to the continuity of *G*. Following [13], we express *β̂* as a new MNC in the space ${X}^{2}$ as

$$\widehat{{\beta}}({M}) = \max \bigl\{ \beta({X}_{1}), \beta({X}_{2}) \bigr\} , $$

where ${X}_{i}$, $i = 1, 2$ denote the natural projections of *M*. Without loss of generality, we take *M*, a nonempty subset of ${X}^{2}$. Following the previous theorem, we have

$$\begin{aligned} \widehat{{\beta}} \bigl(\widehat{{G}}({M}) \bigr) &\leq \widehat{\beta } \bigl({{G}}({X}_{1} \times{X}_{2}) \times{{G}}({X}_{2} \times{X}_{1}) \bigr) \\ &= \max \bigl\{ \beta \bigl({{G}}({X}_{1} \times{X}_{2}) \bigr), \beta \bigl({{G}}({X}_{2} \times {X}_{1}) \bigr) \bigr\} . \end{aligned}$$

Hence, by condition (4.2) and using (2^∘^) of Definition 2.1 we obtain

$$\begin{aligned} &\Psi \bigl(F \bigl(\widehat{{\beta}} \bigl( \widehat{{G}}({M}) \bigr), \varphi \bigl(\widehat{{\beta }} \bigl( \widehat{{G}}({M}) \bigr) \bigr) \bigr) \bigr)\\ &\quad \leq\Psi \left ( \begin{matrix}F(\widehat{\beta}({{G}}({X}_{1} \times{X}_{2}) \times {{G}}({X}_{2} \times{X}_{1})),\\ \varphi(\widehat{\beta}({{G}}({X}_{1} \times{X}_{2}) \times{{G}}({X}_{2} \times{X}_{1})))) \end{matrix} \right ) \\ &\quad = \Psi \left ( \begin{matrix} F(\max\{ \beta({{G}}({X}_{1} \times{X}_{2})), \beta ({{G}}({X}_{2} \times{X}_{1}))\},\\ \varphi(\max\{ \beta({{G}}({X}_{1} \times{X}_{2})), \beta({{G}}({X}_{2} \times{X}_{1}))\})) \end{matrix} \right ) \\ &\quad = \Psi \left ( \max \left \{ \begin{matrix} F(\beta({{G}}({X}_{1} \times{X}_{2})), \varphi(\beta ({{G}}({X}_{1} \times{X}_{2})))),\\ F(\beta({{G}}({X}_{2} \times{X}_{1})), \varphi(\beta({{G}}({X}_{2} \times{X}_{1})))) \end{matrix} \right \} \right ) \\ &\quad \leq\max \left \{ \begin{matrix} \Psi(F(\max\{\beta({X}_{1}),\beta({X}_{2})\}, \varphi(\max\{\beta ({X}_{1}),\beta({X}_{2})\}))),\\ \Psi(F(\max\{\beta({X}_{2}),\beta({X}_{1})\}, \varphi(\max\{\beta ({X}_{2}),\beta({X}_{1})\}))) \end{matrix} \right \} \\ &\quad \leq\max \left \{ \begin{matrix} \Phi(F(\max\{\beta({X}_{1}),\beta({X}_{2})\}, \varphi(\max\{\beta ({X}_{1}),\beta({X}_{2})\}))), \\ \Phi(F(\max\{\beta({X}_{2}),\beta({X}_{1})\}, \varphi(\max\{\beta ({X}_{2}),\beta({X}_{1})\}))) \end{matrix} \right \} \\ &\quad = \Phi \bigl(F \bigl(\max \bigl\{ \beta({X}_{1}), \beta({X}_{2}) \bigr\} , \varphi \bigl(\max \bigl\{ \beta ({X}_{1}),\beta({X}_{2}) \bigr\} \bigr) \bigr) \bigr) \\ &\quad = \Phi \bigl(F \bigl(\widehat{\beta}({M}), \varphi \bigl(\widehat{\beta}({M}) \bigr) \bigr) \bigr), \end{aligned}$$

that is,

$$\begin{aligned} \Psi \bigl(F \bigl(\widehat{\beta} \bigl(\widehat{{G}}({M}) \bigr) \bigr) \bigr) \leq \Phi \bigl(F \bigl(\widehat {\beta}({M})+ \varphi \bigl(\widehat{ \beta}({M}) \bigr) \bigr) \bigr). \end{aligned}$$

Hence, using Theorem 3.3, we conclude that *Ĝ* has at least one fixed point in ${X}^{2}$, and thus *G* has a CFP. □

### Remark 4.4

We can derive some new CFP results from Theorems 4.2-4.3, if we take $F(a,b) = a+b$ with various settings for $\Psi,\Phi$ and *φ*.

## Solvability of an implicit fractional integral equation

Let $C_{+}(I)=C([0,1];\mathbb{R}_{+})$ be the Banach space of all real continuous functions on $I=[0,1]$ equipped with the standard norm

$$ \Vert x \Vert =\max \bigl\{ \bigl\vert x(t) \bigr\vert :t\geq0 \bigr\} . $$

Let *X* be a nonempty and bounded subset of $C_{+}(I)$. Let us define the mapping $\omega:X\times\mathbb{R_{+}}\longrightarrow\mathbb{R_{+}}$ by

$$ \omega(x,\epsilon):=\sup \bigl\{ \bigl\vert x(t)-x(s) \bigr\vert :t,s\in [0,1], \vert t-s \vert \leq\epsilon \bigr\} , \quad x \in X, \epsilon\geq0. $$

Further, let us put

$$ \omega(X,\epsilon):=\sup \bigl\{ \omega(x,\epsilon):x\in X \bigr\} . $$

Let $\mathfrak{X}$ be the set of all nonempty and bounded subsets of $C_{+}(I)$. Then the mapping $\omega_{0}:\mathfrak{X}\longrightarrow \mathbb{R_{+}}$ is defined by

$$\omega_{0}(X):=\lim_{\epsilon\rightarrow0^{+}}\omega(X,\epsilon),\quad X\in \mathfrak{X}. $$

Define

$$ i(x):=\sup \bigl\{ \bigl\vert x(s)-x(t) \bigr\vert - \bigl[x(s)-x(t) \bigr]:t,s \in I,t\leq s \bigr\} $$

and

$$ i(X):=\sup \bigl\{ i(x):x\in X \bigr\} . $$

It is noteworthy that all functions of *X* are nondecreasing on *I* if and only if $i(X)=0$.

Further, we define the function *β* of the family ${\mathfrak {M}}_{C_{+}}(I)$ by the formula

$$ \beta(X):=\omega_{0}(X)+i(X). $$

It has been shown in [14] that the function *β* is a MNC in the space $C_{+}(I)$.

We consider the following assumptions:

$(B_{1})$
the function $a:I\to(0, +\infty)$ is continuous with $M_{1}=\max\{ \vert a(t) \vert :t\in I\}$, nondecreasing, and nonnegative on *I*,
$(B_{2})$
the function $h:I\times\mathbb{R}\to\mathbb{R}$ is continuous in $t,x$ such that $h(I\times\mathbb{R_{+}}) \subseteq\mathbb{R_{+}}$ with $M_{2}=\max\{ \vert h(t,0) \vert :t\in I\}$ and there exists a continuous and nondecreasing function $\varphi:\mathbb{R_{+}}\to \mathbb{R_{+}}$ with $\varphi(0)=0$ and
  5.1 $$\begin{aligned} \bigl\vert h(t,x)-h(t,y) \bigr\vert \leq\sqrt{\varphi \bigl( \vert x-y \vert ^{2} \bigr)} \end{aligned}$$
  for all $t\in I$ and all $x,y\in\mathbb{R}$. Additionally we assume that *φ* is superadditive, *i.e.*, $\varphi(t)+\varphi (s)\leq\varphi(t+s)$ for all $t,s\in\mathbb{R_{+}}$,
$(B_{3})$
the superposition operator *H* generated by the function $h(t,x)$ satisfies for any nonnegative function *x* the condition
  $$\begin{aligned} i(Hx)\leq \sqrt{\Phi \bigl(i(x) \bigr)}, \end{aligned}$$
  where $\Phi:\mathbb{R_{+}}\to \mathbb{R_{+}}$ with $\Phi(t)=\varphi(t^{2})$ is the same function as in $(B_{2})$ and Theorem 3.4,
$(B_{4})$
the function $g:I\times I\times\mathbb{R}\to\mathbb {R}$ is continuous such that $g(I\times I\times\mathbb{R_{+}}) \subseteq\mathbb{R_{+}}$ and
  $$G_{0}=\sup \bigl\{ \bigl\vert g \bigl(t,s,x(s) \bigr) \bigr\vert :t,s\in I, x\in C_{+}(I) \bigr\} < \infty, $$
$(B_{5})$
the function $f:I\to\mathbb{R_{+}}$ is $C_{+}^{1}$ and nondecreasing,
$(B_{6})$
the inequality
  5.2 $$\begin{aligned} M_{1}\Gamma(\gamma+1)+ \bigl(\sqrt{\varphi \bigl(r^{2} \bigr)}+M_{2} \bigr)G_{0} \bigl(f(1)-f(0) \bigr)^{\gamma}\leq\Gamma(\gamma+1) r \end{aligned}$$
  has a positive solution $r_{0}$ such that $\lambda=\frac{\sqrt{2}G_{0}(f(1)-f(0))^{\gamma}}{\Gamma(\gamma+1)}< 1$.

### Theorem 5.1

*Under assumptions* ($B_{1}$)-($B_{6}$), *equation* (1.1) *has a positive solution* $x=x(t)$, *which belongs to the space* $C_{+}(I)$.

### Proof

For $x \in C_{+}(I)$, consider the operators $\mathcal{F}$ and *T* defined on the space $C_{+}(I)$ by the formulas

$$\begin{aligned} &(\mathcal{F}x) (t)=\frac{1}{\Gamma(\gamma)} \int_{0}^{t}\frac {f'(s)}{(f(t)-f(s))^{1-\gamma}}g \bigl(t,s,x(s) \bigr) \,ds, \\ &(Tx) (t)=a(t)+h \bigl(t,x(t) \bigr) (Fx) (t). \end{aligned}$$

Firstly, we prove that $\mathcal{F}$ is self-mapping on $C_{+}(I)$. To this end, it suffices to verity that if $x\in C_{+}(I)$, then $\mathcal{F}x\in C_{+}(I)$. Fix $\epsilon>0$, let $x\in C_{+}(I)$, and let $t_{1},t_{2}\in I$ (without loss of generality assume that $t_{2}\geq t_{1}$) and $\vert t_{2}-t_{1} \vert \leq\epsilon$. Then we get

$$\begin{aligned} &{\Gamma(\gamma)} \bigl\vert (\mathcal{F}x) (t_{2})-(\mathcal{F}x) (t_{1}) \bigr\vert \\ &\quad= \biggl\vert \int_{0}^{t_{2}}\frac{f'(s)}{(f(t_{2})-f(s))^{1-\gamma }}g \bigl(t_{2},s,x(s) \bigr) \,ds- \int_{0}^{t_{1}}\frac{f'(s)}{(f(t_{1})-f(s))^{1-\gamma }}g \bigl(t_{1},s,x(s) \bigr) \,ds \biggr\vert \\ &\quad\leq \biggl\vert \int_{0}^{t_{2}}\frac{f'(s)}{(f(t_{2})-f(s))^{1-\gamma }}g \bigl(t_{2},s,x(s) \bigr) \,ds- \int_{0}^{t_{2}}\frac {f'(s)}{(f(t_{2})-f(s))^{1-\gamma}}g \bigl(t_{1},s,x(s) \bigr) \,ds \biggr\vert \\ &\qquad{}+ \biggl\vert \int_{0}^{t_{2}}\frac{f^{\prime}(s)}{(f(t_{2})-f(s))^{1-\gamma }}g \bigl(t_{1},s,x(s) \bigr) \,ds- \int_{0}^{t_{1}}\frac{f'(s)}{(f(t_{2})-f(s))^{1-\gamma }}g \bigl(t_{1},s,x(s) \bigr) \,ds \biggr\vert \\ &\qquad{}+ \biggl\vert \int_{0}^{t_{1}}\frac{f'(s)}{(f(t_{2})-f(s))^{1-\gamma }}g \bigl(t_{1},s,x(s) \bigr) \,ds- \int_{0}^{t_{1}}\frac{f'(s)}{(f(t_{1})-f(s))^{1-\gamma }}g \bigl(t_{1},s,x(s) \bigr) \,ds \biggr\vert \\ &\quad\leq \int_{0}^{t_{2}}\frac{f'(s)}{(f(t_{2})-f(s))^{1-\gamma }} \bigl\vert g \bigl(t_{2},s,x(s) \bigr)-g \bigl(t_{1},s,x(s) \bigr) \bigr\vert \,ds \\ &\qquad{}+ \int_{t_{1}}^{t_{2}}\frac{f'(s)}{(f(t_{2})-f(s))^{1-\gamma }} \bigl\vert g \bigl(t_{1},s,x(s) \bigr) \bigr\vert \,ds \\ &\qquad{}+ \biggl\vert \int_{0}^{t_{1}} \frac{f'(s)}{(f(t_{2})-f(s))^{1-\gamma}}-\frac {f'(s)}{(f(t_{1})-f(s))^{1-\gamma}} \biggr\vert \bigl\vert g \bigl(t_{1},s,x(s) \bigr) \bigr\vert \,ds. \end{aligned}$$

Therefore, if we denote

$$\begin{aligned} \omega_{g}(\epsilon,\cdot)=\sup \bigl\{ \bigl\vert g(t,s,x)-g \bigl(t',s,x \bigr) \bigr\vert :t,t',s\in I, \bigl\vert t-t' \bigr\vert \leq\epsilon, x \in[-r_{0},r_{0}] \bigr\} , \end{aligned}$$

then

$$\begin{aligned} &{\Gamma(\gamma)} \bigl\vert (\mathcal{F}x) (t_{2})-(\mathcal{F}x) (t_{1}) \bigr\vert \\ &\quad\leq \frac{\omega_{g}(\epsilon,\cdot) }{\gamma} \bigl(f(t_{2})-f(0) \bigr)^{\gamma}+ \frac {G_{0}}{\gamma} \bigl(f(t_{2})-f(t_{1}) \bigr)^{\gamma} \\ &\qquad{}+\frac{G_{0}}{\gamma} \bigl[ \bigl(f(t_{2})-f(t_{0}) \bigr)^{\gamma }- \bigl(f(t_{2})-f(t_{1}) \bigr)^{\gamma}- \bigl(f(t_{1})-f(t_{0}) \bigr)^{\gamma} \bigr] \\ &\quad\leq \frac{\omega_{g}(\epsilon,\cdot) }{\gamma} \bigl(f(t_{2})-f(0) \bigr)^{\gamma}+ \frac {2G_{0}}{\gamma} \bigl(f(t_{2})-f(t_{1}) \bigr)^{\gamma} \\ &\quad\leq \frac{\omega_{g}(\epsilon,\cdot) }{\gamma} \bigl(f(1)-f(0) \bigr)^{\gamma}+ \frac {2G_{0}}{\gamma} \omega(f,\epsilon)^{\gamma}. \end{aligned}$$

Using the notion of uniform continuity of the function *g* on the set $I^{2} \times[-r_{0},r_{0}]$ and *f* on the set *I*, we have $\omega _{g}(\epsilon,\cdot)\longrightarrow0$ and $\omega(f,\epsilon)\longrightarrow 0$ as $\epsilon\longrightarrow0$. Consequently $\mathcal{F}x\in C_{+}(I)$ and thus $Tx\in C_{+}(I)$. Also, we have

5.3 $$\begin{aligned} \bigl\vert (\mathcal{F}x) (t) \bigr\vert &\leq\frac{1}{\Gamma(\gamma)} \int_{0}^{t}\frac {f'(s)}{(f(t)-f(s))^{1-\gamma}} \bigl\vert g \bigl(t,s,x(s) \bigr) \bigr\vert \,ds \\ &\leq\frac{G_{0} }{\Gamma(\gamma)} \int_{0}^{t} \frac{f'(s)}{(f(t)-f(s))^{1-\gamma}}\,ds \\ &\leq \frac{G_{0}(f(1)-f(0))^{\gamma }}{\Gamma(\gamma+1)} \end{aligned}$$

for all $t\in I$. Therefore

$$\begin{aligned} \bigl\vert (Tx) (t) \bigr\vert &\leq \bigl\vert a(t) \bigr\vert + \bigl\vert h(t,x) \bigr\vert \bigl\vert \mathcal{F}x(t) \bigr\vert \\ &\leq M_{1}+ \bigl[ \bigl\vert h(t,x)-h(t,0) \bigr\vert + \bigl\vert h(t,0) \bigr\vert \bigr]\frac{G_{0}(f(1)-f(0))^{\gamma }}{\Gamma(\gamma+1)} \\ &\leq M_{1}+ \bigl(\sqrt{\varphi \bigl( \Vert x \Vert ^{2} \bigr)}+M_{2} \bigr) \frac {G_{0}(f(1)-f(0))^{\gamma}}{\Gamma(\gamma+1)}. \end{aligned}$$

Hence,

$$\begin{aligned} \Vert Tx \Vert \leq M_{1}+ \bigl(\sqrt{\varphi \bigl( \Vert x \Vert ^{2} \bigr)}+M_{2} \bigr)\frac {G_{0}(f(1)-f(0))^{\gamma}}{\Gamma(\gamma+1)}. \end{aligned}$$

Thus, if $\Vert x \Vert \leq r_{0}$ we obtain from assumption $(B_{6})$ the estimate

$$\begin{aligned} \Vert Tx \Vert \leq M_{1}+ \Bigl(\sqrt{\varphi \bigl(r_{0}^{2} \bigr)}+M_{2} \Bigr) \frac {G_{0}(f(1)-f(0))^{\gamma}}{\Gamma(\gamma+1)}\leq r_{0}. \end{aligned}$$

Consequently, the operator *T* maps the ball $B_{r_{0}}\subset C_{+}(I)$ into itself, *i.e.*, $T(B_{r_{0}})\subseteq B_{r_{0}}$, where $B_{r_{0}}=\{x \in C_{+}(I): \Vert x \Vert \leq r_{0}\}$. Therefore, the mapping $T: B_{r_{0}} \rightarrow B_{r_{0}}$ is well defined. Next, what we want to do is to show that the operator *T* is continuous on $B_{r_{0}}$. For this purpose, let $\{x_{n}\}$ be a sequence in $B_{r_{0}}$ such that $x_{n}\to x$. We have to show that $Tx_{n}\to Tx$. In fact, for each $t\in I$, we have

$$\begin{aligned} & \bigl\vert (Tx_{n}) (t)-(Tx) (t) \bigr\vert \\ &\quad= \bigl\vert h \bigl(t,x_{n}(t) \bigr) (\mathcal {F}x_{n}) (t)-h \bigl(t,x(t) \bigr) (\mathcal{F}x) (t) \bigr\vert \\ &\quad\leq \bigl\vert h \bigl(t,x_{n}(t) \bigr) (\mathcal{F}x_{n}) (t)-h \bigl(t,x(t) \bigr) (\mathcal {F}x_{n}) (t) \bigr\vert \\ &\qquad{}+ \bigl\vert h \bigl(t,x(t) \bigr) (\mathcal{F}x_{n}) (t)-h \bigl(t,x(t) \bigr) (\mathcal{F}x) (t) \bigr\vert \\ &\quad\leq \bigl\vert h \bigl(t,x_{n}(t) \bigr)-h \bigl(t,x(t) \bigr) \bigr\vert \bigl\vert (\mathcal{F}x_{n}) (t) \bigr\vert \\ &\qquad{}+ \bigl\vert h \bigl(t,x(t) \bigr) \bigr\vert \bigl\vert ( \mathcal{F}x_{n}) (t)-(\mathcal{F}x) (t) \bigr\vert \\ &\quad\leq\frac{\sqrt{\varphi( \vert x_{n}(t)-x(t) \vert ^{2})}}{\Gamma(\gamma)} \int _{0}^{t}\frac{f'(s)}{(f(t)-f(s))^{1-\gamma}} \bigl\vert g \bigl(t,s,x_{n}(s) \bigr) \bigr\vert \,ds \\ &\qquad{}+ \bigl(\sqrt{\varphi \bigl( \bigl\vert x(t) \bigr\vert ^{2} \bigr)}+M_{2} \bigr)\frac{(f(1)-f(0))^{\gamma}}{\Gamma (\gamma+1)}G_{\epsilon}, \end{aligned}$$

where

$$G_{\epsilon}=\sup \bigl\{ \bigl\vert g(t,s,x)-g(t,s,y) \bigr\vert :t,s \in I, \vert x \vert , \vert y \vert \leq r_{0}, \vert x-y \vert \leq\epsilon \bigr\} . $$

Indeed,

$$\begin{aligned} &\Gamma(\gamma) \bigl\vert (\mathcal{F}x_{n}) (t)-(\mathcal{F}x) (t) \bigr\vert \\ &\quad= \biggl\vert \int_{0}^{t}\frac{f'(s)}{(f(t)-f(s))^{1-\gamma}}g \bigl(t,s,x_{n}(s) \bigr) \,ds- \int _{0}^{t}\frac{f'(s)}{(f(t)-f(s))^{1-\gamma}}g \bigl(t,s,x(s) \bigr) \,ds \biggr\vert \\ &\quad\leq \int_{0}^{t}\frac{f'(s)}{(f(t)-f(s))^{1-\gamma }} \bigl\vert g \bigl(t,s,x_{n}(s) \bigr)-g \bigl(t,s,x(s) \bigr) \bigr\vert \,ds \\ &\quad\leq\frac{(f(1)-f(0))^{\gamma}}{\gamma}G_{\epsilon}. \end{aligned}$$

It follows that

$$\begin{aligned} \Vert Tx_{n}-Tx \Vert \leq \frac{\sqrt{\varphi( \Vert x_{n}-x \Vert ^{2})}}{\Gamma(\gamma +1)} \bigl(f(1)-f(0) \bigr)^{\gamma}G_{0}+ \bigl( \sqrt{\varphi \bigl( \Vert x \Vert ^{2} \bigr)}+M_{2} \bigr)\frac {(f(1)-f(0))^{\gamma}}{\Gamma(\gamma+1)}G_{\epsilon}. \end{aligned}$$

Note that, from the uniform continuity of the function *g* in $I\times I\times[-r_{0},r_{0}]$, it is clear that $\lim_{\epsilon\rightarrow 0^{+}}G_{\epsilon}=0$. As consequence, we have

$$\begin{aligned} \Vert Tx_{n}-Tx \Vert \leq{}& \frac{\sqrt{\varphi( \Vert x_{n}-x \Vert ^{2})}}{\Gamma(\gamma +1)} \bigl(f(1)-f(0) \bigr)^{\gamma}G_{0}+ \bigl( \sqrt{\varphi \bigl( \Vert x \Vert ^{2} \bigr)}+M_{2} \bigr)\\ &{}\times \frac {(f(1)-f(0))^{\gamma}}{\Gamma(\gamma+1)}G_{\epsilon}\rightarrow0 \quad\mbox{as } \epsilon \rightarrow0^{+}. \end{aligned}$$

This proves that *T* is continuous on $B_{r_{0}}$. Consider the operator *T* on the subset $B^{+}_{r_{0}} $ of the ball $B_{r_{0}}$ defined in the following way:

$$\begin{aligned} B^{+}_{r_{0}}= \bigl\{ x\in B_{r_{0}}:x(t)\geq0, \mbox{for } t \in I \bigr\} . \end{aligned}$$

Obviously, the set $B^{+}_{r_{0}} $ is nonempty, bounded, closed, and convex. In view of our assumptions, if $x(t)\geq0$, then $(Tx)(t)\geq0$ for all $t\in I$. Thus *T* transforms the set $B^{+}_{r_{0}} $ into itself. Moreover, *T* is continuous on $B^{+}_{r_{0}}$. Let *X* be a nonempty subset of $B^{+}_{r_{0}}$. Fix $\epsilon>0$ and $t_{1},t_{2}\in I$ with $\vert t_{2}-t_{1} \vert \leq\epsilon$. Without loss of generality we assume that $t_{2}\geq t_{1}$. Then we get

5.4 $$\begin{aligned} & \bigl\vert (Tx) (t_{2})-(Tx) (t_{1}) \bigr\vert \\ &\quad= \bigl\vert a(t_{2})+h \bigl(t_{2},x(t_{2}) \bigr) (\mathcal {F}x) (t_{2})-a(t_{1})-h \bigl(t_{1},x(t_{1}) \bigr) (\mathcal{F}x) (t_{1}) \bigr\vert \\ &\quad\leq \bigl\vert a(t_{2})-a(t_{1}) \bigr\vert + \bigl\vert h \bigl(t_{2},x(t_{2}) \bigr) (\mathcal {F}x) (t_{2})-h \bigl(t_{1},x(t_{2}) \bigr) ( \mathcal{F}x) (t_{2}) \bigr\vert \\ &\qquad{}+ \bigl\vert h \bigl(t_{1},x(t_{2}) \bigr) ( \mathcal{F}x) (t_{2})-h \bigl(t_{1},x(t_{1}) \bigr) (\mathcal {F}x) (t_{2}) \bigr\vert \\ &\qquad{}+ \bigl\vert h \bigl(t_{1},x(t_{1}) \bigr) ( \mathcal{F}x) (t_{2})-h \bigl(t_{1},x(t_{1}) \bigr) (\mathcal {F}x) (t_{1}) \bigr\vert \\ &\quad\leq \bigl\vert a(t_{2})-a(t_{1}) \bigr\vert + \bigl\vert h \bigl(t_{2},x(t_{2}) \bigr)-h \bigl(t_{1},x(t_{2}) \bigr) \bigr\vert \bigl\vert ( \mathcal {F}x) (t_{2}) \bigr\vert \\ &\qquad{}+ \bigl\vert h \bigl(t_{1},x(t_{2}) \bigr)-h \bigl(t_{1},x(t_{1}) \bigr) \bigr\vert \bigl\vert ( \mathcal{F}x) (t_{2}) \bigr\vert \\ &\qquad{}+ \bigl\vert h \bigl(t_{1},x(t_{1}) \bigr) \bigr\vert \bigl\vert (\mathcal{F}x) (t_{2})-(\mathcal{F}x) (t_{1}) \bigr\vert \\ &\quad\leq\omega(a,\epsilon)+ \bigl[\zeta_{r_{0}}(h,\epsilon)+\sqrt{\varphi \bigl( \bigl\vert x(t_{2})-x(t_{1}) \bigr\vert ^{2} \bigr)} \bigr]\frac{G_{0}(f(t_{2})-f(0))^{\gamma}}{\Gamma (\gamma+1)} \\ &\qquad{}+ \bigl(\sqrt{\varphi \bigl( \Vert x \Vert ^{2} \bigr)}+M_{2} \bigr) \biggl[\frac{\omega_{g}(\epsilon,\cdot) }{\gamma} \bigl(f(1)-f(0) \bigr)^{\gamma}+\frac{2G_{0}}{\gamma } \bigl(f(t_{2})-f(t_{1}) \bigr)^{\gamma} \biggr], \end{aligned}$$

where

$$\begin{aligned} \zeta_{r_{0}}(h,\epsilon)=\sup \bigl\{ \bigl\vert h(t,x)-h \bigl(t',x \bigr) \bigr\vert :t,t'\in I,x \in[0,r_{0}], \bigl\vert t-t' \bigr\vert \leq\epsilon \bigr\} . \end{aligned}$$

By means of the mean value theorem on $[t_{1},t_{2}]$, we get

5.5 $$\begin{aligned} \bigl\vert f(t_{2})-f(t_{1}) \bigr\vert ^{\gamma}\leq m^{\gamma} \vert t_{2}-t_{1} \vert ^{\gamma}. \end{aligned}$$

Now, plugging equation (5.5) into (5.4), we get

$$\begin{aligned} & \bigl\vert (Tx) (t_{2})-(Tx) (t_{1}) \bigr\vert \\ &\quad\leq \omega(a,\epsilon)+ \bigl[ \zeta_{r_{0}}(h,\epsilon)+\sqrt{\varphi \bigl( \bigl\vert x(t_{2})-x(t_{1}) \bigr\vert ^{2} \bigr)} \bigr] \frac{G_{0}(f(1)-f(0))^{\gamma}}{\Gamma (\gamma+1)} \\ &\qquad{}+ \bigl(\sqrt{\varphi \bigl( \Vert x \Vert ^{2} \bigr)}+M_{2} \bigr) \biggl[\frac{\omega_{g}(\epsilon,\cdot) }{\gamma} \bigl(f(1)-f(0) \bigr)^{\gamma}+\frac{2G_{0}}{\gamma}(m\epsilon)^{\gamma} \biggr], \end{aligned}$$

hence,

$$\begin{aligned} \omega(Tx,\epsilon)\leq{}& \omega(a,\epsilon)+ \bigl[ \zeta_{r_{0}}(h,\epsilon)+\sqrt{\varphi \bigl(\omega ^{2}(x, \epsilon) \bigr)} \bigr]\frac{G_{0}(f(1)-f(0))^{\gamma}}{\Gamma(\gamma+1)} \\ &{}+ \Bigl(\sqrt{\varphi \bigl(r_{0}^{2} \bigr)}+M_{2} \Bigr) \biggl[\frac{\omega_{g}(\epsilon,\cdot) }{\gamma} \bigl(f(1)-f(0) \bigr)^{\gamma}+\frac{2G_{0}}{\gamma}(m\epsilon)^{\gamma} \biggr]. \end{aligned}$$

Thus, taking the supremum on *X*, we obtain

$$\begin{aligned} \omega(TX,\epsilon)\leq{}& \omega(a,\epsilon)+ \bigl[ \zeta_{r_{0}}(h,\epsilon)+\sqrt{\varphi \bigl(\omega ^{2}(X, \epsilon) \bigr)} \bigr]\frac{G_{0}(f(1)-f(0))^{\gamma}}{\Gamma(\gamma+1)} \\ &{}+ \Bigl(\sqrt{\varphi \bigl(r_{0}^{2} \bigr)}+M_{2} \Bigr) \biggl[\frac{\omega_{g}(\epsilon,\cdot) }{\gamma} \bigl(f(1)-f(0) \bigr)^{\gamma}+\frac{2G_{0}}{\gamma}(m\epsilon)^{\gamma} \biggr]. \end{aligned}$$

From the uniform continuity of the function *g* on the set $I\times I\times\mathbb{R_{+}}$ and *h* on the set $I \times [0,r_{0}]$ and the continuity of the function *a* on *I*, we have $\omega_{g}(\epsilon,\cdot) \to0$, $\zeta_{r_{0}}(h,\epsilon) \to0$ and $\omega(a,\epsilon) \to0$ as $\epsilon\to0$. So we let $\epsilon\to0$ to obtain

5.6 $$\begin{aligned} \omega_{0}(TX)\leq \frac{G_{0}(f(1)-f(0))^{\gamma}}{\Gamma(\gamma+1)} \sqrt{ \varphi \bigl(\omega _{0}^{2}(X) \bigr)}\leq\lambda \sqrt {\varphi \bigl(\omega _{0}^{2}(X) \bigr)}\leq\sqrt {\varphi \bigl(\omega_{0}^{2}(X) \bigr)}. \end{aligned}$$

Let $x\in X$ and $t_{1},t_{2}\in I$ with $t_{1}< t_{2}$. Then

$$\begin{aligned} & \bigl\vert (Tx) (t_{2})-(Tx) (t_{1}) \bigr\vert - \bigl[(Tx) (t_{2})-(Tx) (t_{1}) \bigr] \\ &\quad= \bigl\vert a(t_{2})+h \bigl(t_{2},x(t_{2}) \bigr) (\mathcal {F}x) (t_{2})-a(t_{1})-h \bigl(t_{1},x(t_{1}) \bigr) (\mathcal{F}x) (t_{1}) \bigr\vert \\ &\qquad{}- \bigl[a(t_{2})+h \bigl(t_{2},x(t_{2}) \bigr) ( \mathcal {F}x) (t_{2})-a(t_{1})-h \bigl(t_{1},x(t_{1}) \bigr) (\mathcal{F}x) (t_{1}) \bigr] \\ &\quad\leq \bigl\{ \bigl\vert a(t_{2})-a(t_{1}) \bigr\vert - \bigl[a(t_{2})-a(t_{1}) \bigr] \bigr\} \\ &\qquad{}+ \bigl\vert h \bigl(t_{2},x(t_{2}) \bigr) (\mathcal{F}x) (t_{2}) -h \bigl(t_{1},x(t_{1}) \bigr) ( \mathcal{F}x) (t_{2}) \bigr\vert \\ &\qquad{}+ \bigl\vert h \bigl(t_{1},x(t_{1}) \bigr) (\mathcal {F}x) (t_{2})-h \bigl(t_{1},x(t_{1}) \bigr) ( \mathcal{F}x) (t_{1}) \bigr\vert \\ &\qquad{}- \bigl[h \bigl(t_{2},x(t_{2}) \bigr) (\mathcal{F}x) (t_{2})-h \bigl(t_{1},x(t_{1}) \bigr) (\mathcal {F}x) (t_{2}) \bigr] \\ &\qquad{}+ \bigl[h \bigl(t_{1},x(t_{1}) \bigr) (\mathcal{F}x) (t_{2})-h \bigl(t_{1},x(t_{1}) \bigr) (\mathcal {F}x) (t_{1}) \bigr] \\ &\quad\leq \bigl\{ \bigl\vert h \bigl(t_{2},x(t_{2}) \bigr)-h \bigl(t_{1},x(t_{1}) \bigr) \bigr\vert - \bigl[h \bigl(t_{2},x(t_{2}) \bigr)-h \bigl(t_{1},x(t_{1}) \bigr) \bigr] \bigr\} (\mathcal{F}x) (t_{2}) \\ &\qquad{}+h \bigl(t_{1},x(t_{1}) \bigr) \bigl\{ \bigl\vert ( \mathcal{F}x) (t_{2})-(\mathcal {F}x) (t_{1}) \bigr\vert - \bigl[(\mathcal{F}x) (t_{2})-(\mathcal{F}x) (t_{1}) \bigr] \bigr\} \\ &\quad\leq i(Hx) \frac{G_{0}(f(1)-f(0))^{\gamma}}{\Gamma(\gamma+1)}. \end{aligned}$$

From the above estimate and assumption $(B_{6})$, we conclude that

$$\begin{aligned} i(Tx)\leq\frac{G_{0}(f(1)-f(0))^{\gamma}}{\Gamma(\gamma+1)}\sqrt{\varphi \bigl(i^{2}(x) \bigr)}\leq \sqrt{\varphi \bigl(i^{2}(x) \bigr)} \end{aligned}$$

and

5.7 $$\begin{aligned} i(TX)\leq\frac{G_{0}(f(1)-f(0))^{\gamma}}{\Gamma(\gamma+1)}\sqrt{\varphi \bigl(i^{2}(X) \bigr)}\leq\sqrt{\varphi \bigl(i^{2}(X) \bigr)}. \end{aligned}$$

From (5.6), (5.7) and the definition of the MNC *β*, we obtain

$$\begin{aligned} \beta(TX)&=\omega_{0}(TX)+i(TX)\leq \sqrt {\varphi \bigl(\omega_{0}^{2}(X) \bigr)}+\sqrt{ \varphi \bigl(i^{2}(X) \bigr)} \\ &\leq \frac{G_{0}(f(1)-f(0))^{\gamma}}{\Gamma(\gamma+1)} \Bigl(\sqrt{\varphi \bigl(\omega _{0}^{2}(X) \bigr)}+\sqrt{\varphi \bigl(i^{2}(X) \bigr)} \Bigr) \\ &\leq\frac{\sqrt{2}G_{0}(f(1)-f(0))^{\gamma}}{\Gamma(\gamma+1)}\sqrt {\varphi \bigl(\omega_{0}^{2}(X)+i^{2}(X) \bigr)} \\ &\leq\sqrt{\varphi \bigl(\omega_{0}(X)+i(X) \bigr)^{2}} \leq \sqrt{\varphi \bigl(\beta^{2}(X) \bigr)}. \end{aligned}$$

Now, by considering the functions $\Psi,\Phi:[0,\infty) \to[0,\infty)$ defined by

$$\Psi(t)=t^{2} \quad\mbox{and}\quad \Phi(t)=\varphi \bigl(t^{2} \bigr), $$

we get

$$\Psi\bigl(\beta \bigl(T(X) \bigr)\bigr)\leq\Phi \bigl(\beta(X) \bigr). $$

Thus, we obtain Theorem 3.4. □

Finally, we present an illustrative example for Theorem 5.1.

### Example

Consider the integral equation of the form

5.8 $$ x(t)=\frac{1}{2} +t^{2} + \frac{2tx(t)}{3(1+t)\Gamma(\frac{1}{2})} \int _{0}^{t}\frac{2s}{\sqrt{t^{2}-s^{2}}}\frac{t}{(1+s^{2})(1+x^{2}(s))} \,ds, \quad 0\leq t\leq1. $$

Observe that equation (5.8) is a special case of equation (1.1). In this example, we have

$$\begin{aligned} a(t)=\frac{1}{2} + t^{2},\qquad f(t)=t^{2},\qquad g(t,s,x)= \frac {t}{4(1+s^{2})(1+x^{2})},\qquad h(t,x)=\frac{8tx}{1+t}. \end{aligned}$$

Let us check that all the assumptions of Theorem 5.1 are satisfied:

- Assumption $(B_{1})$. It is trivial and $M_{1}=\frac{3}{2}$.
- Assumption $(B_{2})$. For $t \in I$ and $x,y \in\mathbb{R}$, we have
  $$\begin{aligned} \bigl\vert h(t,x)-h(t,y) \bigr\vert &\leq \biggl\vert \frac{8tx}{1+t}- \frac{8ty}{1+t} \biggr\vert =\frac {8t}{1+t} \vert x-y \vert \leq \sqrt{2 \vert x-y \vert ^{2}}=\sqrt{\varphi \bigl( \vert x-y \vert ^{2} \bigr)}, \end{aligned}$$
  where $\varphi(t)=2t, t \geq0$. So assumption $(B_{2})$ is satisfied with $M_{2}=0$.
- Assumption $(B_{3})$. It is trivial. Indeed, taking an arbitrary nonnegative function $x\in C_{+}(I)$ and $t_{1},t_{2}\in I$ such that $t_{1}\leq t_{2}$, we obtain
  $$\begin{aligned} & \bigl\vert (Hx) (t_{2})-(Hx) (t_{1}) \bigr\vert - \bigl[(Hx) (t_{2})-(Hx) (t_{1}) \bigr] \\ &\quad= \bigl\vert h \bigl(t_{2},x(t_{2}) \bigr)-h \bigl(t_{1},x(t_{1}) \bigr) \bigr\vert - \bigl[h \bigl(t_{2},x(t_{2}) \bigr)-h \bigl(t_{1},x(t_{1}) \bigr) \bigr] \\ &\quad= \biggl\vert \frac{8t_{2}x(t_{2})}{(1+t_{2})}-\frac {8t_{1}x(t_{1})}{(1+t_{1})} \biggr\vert - \biggl[ \frac{8t_{2}x(t_{2})}{(1+t_{2})}-\frac {8t_{1}x(t_{1})}{(1+t_{1})} \biggr] \\ &\quad\leq \biggl\vert \frac{8t_{2}x(t_{2})}{(1+t_{2})}-\frac {8t_{2}x(t_{1})}{(1+t_{2})} \biggr\vert + \biggl\vert \frac{8t_{2}x(t_{1})}{(1+t_{2})}-\frac {8t_{1}x(t_{1})}{(1+t_{1})} \biggr\vert \\ &\qquad{}- \biggl[\frac{8t_{2}x(t_{2})}{(1+t_{2})}-\frac {8t_{2}x(t_{1})}{(1+t_{2})}+\frac{8t_{2}x(t_{1})}{(1+t_{2})}- \frac {8t_{1}x(t_{1})}{(1+t_{1})} \biggr] \\ &\quad\leq\frac{8t_{2}}{(1+t_{2})} \bigl\vert x(t_{2})-x(t_{1}) \bigr\vert + \biggl\vert \frac {8t_{2}}{(1+t_{2})}-\frac{8t_{1}}{(1+t_{1})} \biggr\vert x(t_{1}) \\ &\qquad{}-\frac{8t_{2}}{(1+t_{2})} \bigl[x(t_{2})-x(t_{1}) \bigr]- \biggl[ \frac {8t_{2}}{(1+t_{2})}-\frac{8t_{1}}{(1+t_{1})} \biggr]x(t_{1}) \\ &\quad\leq\frac{8t_{2}}{(1+t_{2})} \bigl\{ \bigl\vert x(t_{2})-x(t_{1}) \bigr\vert - \bigl[x(t_{2})-x(t_{1}) \bigr] \bigr\} \\ &\quad\leq\frac{8t_{2}}{(1+t_{2})}i(x)\leq4 i(x)=\sqrt{2i^{2}(x)}=\sqrt {\varphi \bigl(i^{2}(x) \bigr)} \\ &\quad\leq\sqrt{\Phi \bigl(i(x) \bigr)}. \end{aligned}$$
- Assumption $(B_{4})$. It is trivial with $G_{0}\leq\frac{1}{4}$.
- Assumption $(B_{5})$. It is trivial.
- Assumption $(B_{6})$. In this case inequality (5.2) has the form
  $$\begin{aligned} \frac{3}{2}\Gamma \biggl(\frac{1}{2}+1 \biggr)+\sqrt{ \varphi \bigl(r_{0}^{2} \bigr)}\frac {1}{4}\leq\Gamma \biggl(\frac{1}{2}+1 \biggr) r_{0} \end{aligned}$$
  or
  $$\begin{aligned} \frac{3}{4}\Gamma \biggl(\frac{1}{2} \biggr)+\frac{\sqrt{2}}{4}r_{0} \leq \frac {1}{2}\Gamma \biggl(\frac{1}{2} \biggr) r_{0}, \end{aligned}$$
  and this admits
  $$\begin{aligned} r_{0}=\frac{3\Gamma(\frac{1}{2})}{2\Gamma(\frac{1}{2})-\sqrt{2}} \end{aligned}$$
  as a positive solution since $\Gamma(\frac{1}{2})=1.77245$.

Thus, from all the above mentioned observations, it is clear that equation (5.8) satisfies all the requirements of Theorem 5.1 and hence, the functional integral equation (5.8) has a positive solution in $C_{+}(I)$.

## Conclusions

In this work we studied the problem of the existence of positive solutions of fractional integral equations by means of the MNC in the association with DFPT. For this purpose we first established a new DFPT and its coupled version that generalized some existing results. To support our results, an illustrative example is provided.
